# E2 Tyrosine 102 Regulates MmuPV1 Pathogenesis In Vivo

**DOI:** 10.3390/pathogens14090913

**Published:** 2025-09-11

**Authors:** Jessica Gonzalez, Marsha DeSmet, Kennedy Stoll, Leny Jose, Neil Christensen, Elliot J. Androphy

**Affiliations:** 1 Department of Microbiology and Immunology, Indiana University School of Medicine, Indianapolis, IN 46202, USA; jkaygonzalez@gmail.com (J.G.); mdesmet@iu.edu (M.D.); kenstoll@iu.edu (K.S.); 2 Department of Dermatology, Indiana University School of Medicine, Indianapolis, IN 46202, USA; leny@rgcb.res.in; 3 Department of Pathology, College of Medicine, Pennsylvania State University, 500 University Drive, Hershey, PA 17033, USA; ndc1@psu.edu

**Keywords:** MmuPV1, replication, tyrosine phosphorylation, E2

## Abstract

The papillomavirus (PV) life cycle is strictly controlled and can be divided into the following three distinct stages: initial infection, maintenance, and amplification. The papillomavirus E2 gene encodes a multifunctional protein responsible for regulating transcription and replication by recruiting viral and host factors to the viral DNA genome. Our lab has previously reported that tyrosine 102 may impact bovine (BPV) and human (HPV) viral replication in cell culture systems. This tyrosine is conserved in the E2 protein of the murine papillomavirus MmuPV1. To investigate how this amino acid impacts the MmuPV1 lifecycle in vivo, we generated potential phosphorylation mimetic (Y102E) and phosphorylation deficient (Y102F) mutants in the E2 open reading frame. The Y102F mutant protein supported both transcriptional activation and transient replication, while Y102E was defective. However, Y102E was capable of associating with E1 and the Brd4 C-terminal motif. When these E2-mutated MmuPV1 genomes were introduced into the skin of immunocompromised mice, only Y102F was capable of inducing papilloma development and production of infectious progeny virus. These findings demonstrate that while highly conserved, tyrosine at this position is not required by the virus. These data suggest that the chemical nature of the amino acid at this position can influence E2 activity and viral replication.

## 1. Introduction

Papillomaviruses (PVs) are epitheliotropic, covalently closed double-stranded DNA viruses with genomes that are approximately eight kilobases in length. The genome organization is similar across PV types, and several ORFs are highly conserved, including those of the replication proteins E1 and E2 and the major and minor capsid proteins L1 and L2. E1 and E2 proteins are present at low levels in cells, and most experimental characterizations of their properties depend on heterologous overexpression systems. The full replicative program of papillomaviruses is strictly tied to epithelial differentiation and can be divided into three distinct stages in vitro: establishment, maintenance, and vegetative amplification [1]. Whether these same three discrete stages of genome replication occur in natural in vivo infections is not clear.

Infection occurs when a break in the epithelium allows virion access to the basement membrane, where it can bind and enter basal cells [2]. The virion is then trafficked in a retrograde fashion to the trans-Golgi network and the endoplasmic reticulum [3,4] where it will remain until the cell enters mitosis, when nuclear envelope breakdown permits the virion to gain access to the nucleus [5]. Inside the nucleus, the early viral proteins E1 and E2 are expressed, and the virus undergoes an initial burst of genome replication to a low copy number of unintegrated episomes.

After the virus has successfully established infection in the nucleus, it enters the maintenance phase, in which the virus replicates in synchrony with host cells to maintain a reservoir of nuclear episomes as infected cells divide [6]. During this stage, replicated viral genomes must be partitioned to the new daughter cells. The process of viral genome segregation to daughter cells is mediated by E2 association with mitotic chromosomes [7,8]. As the infected host cell differentiates and moves upward through the stratified epithelium, the virus enters the vegetative amplification stage, in which the capsid proteins L1 and L2 are highly expressed, and the viral genome is replicated to a high copy number. Nascent virions are assembled in the uppermost layer of the epithelium and shed during the process of desquamation. The potential roles of E2 in vegetative replication and virion production are unknown.

The viral protein E2 is the primary regulator of the PV life cycle, responsible for orchestrating viral gene expression and the replication and partitioning of viral genomes; post-translational modifications (PTMs) have been implicated in regulating E2 function. One residue of interest is Y102, found in the transactivation domain (TAD) of multiple PV types, including BPV-1, HPV-16, HPV-31, and the murine papillomavirus MmuPV1.

The aim of this study was to define the functional role of residue 102 in MmuPV1 E2 and determine how its modification influences viral transcription, replication, and pathogenic outcomes in vivo. Here we present evidence that the identity of residue 102 in MmuPV1 E2 contributes to regulation of viral transcription and replication through alteration of E2 interactions with cellular proteins. We demonstrate that Y102E is incapable of activating transcription or supporting transient replication and fails to produce cutaneous lesions in vivo. In contrast, the Y102F is fully functional in vitro and capable of inducing proliferative wart formation in vivo. Together, these findings suggest that phosphorylation on tyrosine 102 may represent an inhibitory E2 phenotype that may limit viral replication and prevent cell lysis.

## 2. Materials and Methods

Plasmids and mutagenesis. The MusPV1 genome (pMusPV) was kindly provided by John Schiller. To construct pUC19-E2, the E2 open reading frame was excised from pMusPV using the restriction enzyme KpnI and cloned into the pUC19 backbone. Site-directed mutagenesis was then carried out with the Q5 kit (New England Biolabs, Ipswich, MA, USA; Cat. #M0492S), using the following mutagenic primers: Y102E Fwd, CCGTGAGATGGAAGACAGCACTG; Y102E Rev, CTAGTTTCAGCCATTGTCC; Y102F Fwd, CGTGAGATGTTTGACAGCACTG; Y102F Rev, GCTAGTTTCAGCCATTGTC; E2 TTL Fwd, ATACTATATTaGGAGCTTGTTAGGAAAG; and E2 TTL Rev, GTGGTCCGCTAAACATTTAC. Verified E2 mutant fragments were subsequently reintroduced into the pMusPV backbone to generate full-length viral genomes. For expression studies, Y102 mutant E2 was inserted into the pCI-V5 vector using the primers V5 E2 F (ATGCGAATTCATGAACAGCCTGGAAACACGTTT) and V5 E2 R (ATGCGTCGACTCAGAGTCCGTCTAAGAAGC), yielding pCI-V5-E2. The plasmid pCN-GST:hBrd4(1224–1362) was employed in GST-CTM co-immunoprecipitation assays [9]. The constructs WT pCI-V5-E2, pCI-V5-E2 TTL, pCI-myc-E1, pCI-myc-E1 TTL, and mFLori have been described previously [10].

Cell culture and transfection. Cells were maintained under standard conditions of 37 °C in a humidified atmosphere containing 5% CO_2_. The HEK293TT, HEK293TTF, CV-1, and C33-A lines were propagated in Dulbecco’s Modified Eagle Medium (DMEM; Life Technologies, Carlsbad, CA, USA) supplemented with 10% fetal bovine serum (FBS; Peak Serum, CO, USA) and 1% penicillin–streptomycin (Life Technologies). For DNA delivery, cells were transfected either with Lipofectamine 2000 (Invitrogen, Carlsbad, CA, USA) according to the manufacturer’s protocol or with polyethylenimine (PEI) at a concentration of 2 µg/mL, using a PEI:DNA mass ratio of 2:1.

Transcription activation assay. C33-A cells were co-transfected with equal amounts of either wild-type or Y102 mutant V5-E2 plasmids along with the E2-responsive firefly luciferase reporter construct (pGL2-E2BS-Luc). At ~48 h after transfection, cells were harvested and lysed, and luciferase activity was quantified using the Luciferase Assay System (Promega, Madison, WI, USA). Luminescence was read on a PHERAStar plate reader, and reporter activity was normalized against control samples transfected with the reporter alone. Statistical significance between wild-type and mutant groups was evaluated using a paired *t*-test. For overexpression experiments, C33-A cells received graded amounts of V5-E2 plasmid DNA, while the total DNA concentration across samples was equalized by supplementing with the empty vector pCI-V5.

Transient DNA replication assay. Transient replication experiments were carried out following established protocols [11]. C33-A cells were plated in 96-well format and co-transfected with the mFLori plasmid, the Renilla luciferase control vector (pRLuc), and expression constructs encoding either wild-type or mutant V5-E2 together with wild-type or TTL E1. Seventy-two hours after transfection, replication activity was evaluated using the Dual-Glo Luciferase Assay System (Promega). Luminescence was recorded with a PHERAStar plate reader, and data were expressed as the ratio of firefly to Renilla luciferase signals. Values were further normalized to the baseline obtained from samples containing only reporter plasmids without E1 or E2. Each experiment was repeated at least three independent times, with eight technical replicates per condition. Statistical significance was assessed by paired *t*-tests comparing groups with versus without E1.

Immunofluorescence. CV-1 cells were seeded onto glass coverslips placed in six-well plates and transfected with expression plasmids encoding WT V5-E2, Y102F V5-E2, or Y102E V5-E2. Untransfected cells served as negative controls. At ~48 h post-transfection, cells were fixed in 4% paraformaldehyde. Membranes were permeabilized for 10 min at room temperature in PBS containing 0.1% Triton X-100, followed by blocking for 1 h in PBS with 0.1% Triton X-100 supplemented with 10% normal goat serum. Samples were incubated overnight at 4 °C with anti-V5 antibody (Cell Signaling Technology, Danvers, MA, USA; clone D3H8Q) diluted 1:1200. After primary antibody removal, cells were washed three times with PBS and incubated for ~2 h at room temperature, protected from light, with Alexa Fluor 594–conjugated goat anti-rabbit IgG (Invitrogen, #A-11012) at a dilution of 1:3000. Coverslips were washed three additional times in PBS and mounted using ProLong Gold antifade reagent with DAPI (Invitrogen).

DNA isolation and PCR. Genomic DNA was extracted from tissue specimens using the DNeasy Blood and Tissue Kit (Qiagen, Hilden, Germany). PCR amplification of MmuPV1 genomic regions was performed with the following primers: m1800 Fwd (TGTTGGGTCATCATCGTTGT), m3300 Rev (GGAAGTTTGCAATAACCTTCCAGTCCC), m3000 Fwd (GTAGTATGGTGCAGTGGGCA), and mE2-L2 Rev (ATCGGGGGTCACCTCAAG). PCR products were purified using the GFX PCR DNA and Gel Band Purification Kit (Cytiva, Marlborough, MA, USA), and sequencing was carried out to verify the presence of the Y102F mutation in the tissue-derived DNA.

Immunoprecipitation and immunoblotting. For CTM co-immunoprecipitation, HEK293TT cells were transfected with plasmids encoding WT or mutant V5-E2 together with GST:Brd4-CTM. At ~48 h post-transfection, cells were rinsed with cold PBS and lysed for 30 min on ice in IP lysis buffer (50 mM Tris, pH 7.2; 150 mM NaCl; 0.1% NP-40; 1 mM DTT; 1× protease inhibitor cocktail). Lysates were collected by scraping, clarified at 16,000× *g* for 30 min at 4 °C, and ~5–10% of each sample was reserved as input. The remainder was incubated overnight at 4 °C with V5 magnetic beads (MBL #M167-11, Carlsbad, CA, USA) or custom antibodies against MmuPV1 E2. Following three washes in IP lysis buffer, bound proteins were eluted in 1× Laemmli buffer at 65 °C for 5 min. For some experiments, cells expressing V5-E2 Y102E were treated with 50 µM MG-132 (Sigma-Aldrich, C2211, St. Louis, MO, USA) for 5 h before harvest.

For E1 co-immunoprecipitation, HEK293TT cells were transfected with V5-E2 and myc-E1 expression vectors. Twenty-four hours later, one set of Y102E V5-E2/myc-E1 transfected cells was exposed to 1 µM MG-132 (Sigma-Aldrich, #M7449) for 4.5 h. After treatment, cells were processed as described above. Supernatants were incubated overnight at 4 °C with myc-conjugated magnetic beads (MedChemExpress, Monmouth Junction, NJ, USA; #HY-K0206) that had been pre-blocked with 2% BSA and rinsed in PBS. Beads were subsequently washed five times in IP wash buffer (50 mM Tris, pH 7.5; 170 mM NaCl; 0.1% NP-40; 1× protease inhibitor cocktail), once in IP lysis buffer, and eluted in 1× Laemmli buffer by heating to 65 °C.

For detection of MmuPV1 E2 in wart tissue, frozen lesions were disrupted with a sonicator (5–10 pulses, 10 s each) in IP lysis buffer. Lysates were clarified by centrifugation at 15,000 rpm for 10 min. From 400 to 600 µg total protein, E2 was immunoprecipitated overnight at 4 °C using MmuPV1 E2 antibodies with magnetic beads (Active Motif #53014, Carlsbad, CA, USA). After three washes in IP lysis buffer, protein complexes were released by incubation in 1× Laemmli buffer at 65 °C for 5 min.

Sample electrophoresis and Western blotting. Immunoprecipitated complexes were resolved by SDS-PAGE on ExpressPlus 4–12% polyacrylamide gels (Bio-Rad, Hercules, CA, USA) and transferred to 0.45 µm PVDF membranes (Millipore, Boston, MA, USA) using semi-dry transfer. Membranes were blocked with 5% non-fat milk in PBST and washed at least three times in PBST prior to primary antibody incubation (overnight at 4 °C, diluted in PBST). Detection was performed with the indicated primary antibodies followed by appropriate secondary antibodies. Blots were visualized using SuperSignal West Femto Maximum Sensitivity chemiluminescent substrate (Thermo Scientific, Waltham, MA, USA).

Generation of E2 monoclonal and polyclonal antibodies. Purified his-tagged mouse E2 protein (aa 1-221) was used as the antigen for immunization in a rabbit and in mice. Standard immunizations and hybridoma production followed previously published methods (53). We generated several murine anti-MmuPV1 E2 monoclonal antibodies and tested their ability to detect the MmuPV1 E2 in our cell culture studies. In this study we used monoclonal antibody, P2H7, which was effective for immunoprecipitation and Western blot detection of MmuPV1 E2. Approximately 30 μL of P2H7 hybridoma supernatant was used in each immunoprecipitation (1:33).

In vivo viral DNA challenge. All animal procedures were carried out at the IU School of Medicine Laboratory Animal Resource Center (LARC) under approved IACUC protocol #23035 and in compliance with international standards for animal welfare. Experiments were conducted in homozygous Hsd:Athymic Nude-Foxn1^nu^ mice (Inotiv, West Lafayette, IN, USA). To enhance MmuPV1 copy number, mice were provided drinking water supplemented with estradiol (8 µg/mL) beginning one week prior to injection [12]. Seventy-two hours before viral challenge, animals were anesthetized with isoflurane, and tails were gently pre-wounded using autoclaved sandpaper, as previously described [13,14]. Circularized viral genomes were generated as reported earlier [15], resuspended in TE buffer, and ~18 µg was injected intradermally into the pre-wounded tail site of anesthetized mice. Inoculations included viral DNA containing either the Y102E or Y102F E2 mutation. Mice were examined weekly for lesion development and monitored for 5.5 months following infection.

Statistical analysis. All experiments included at least three independent biological replicates. Data analysis was performed using Microsoft Excel. Statistical significance was generally set at *p* < 0.05 unless otherwise indicated.

## 3. Results

### 3.1. The MmuPV1 E2 Protein Is Phosphorylated at Tyrosine Residues

**Figure 1:**
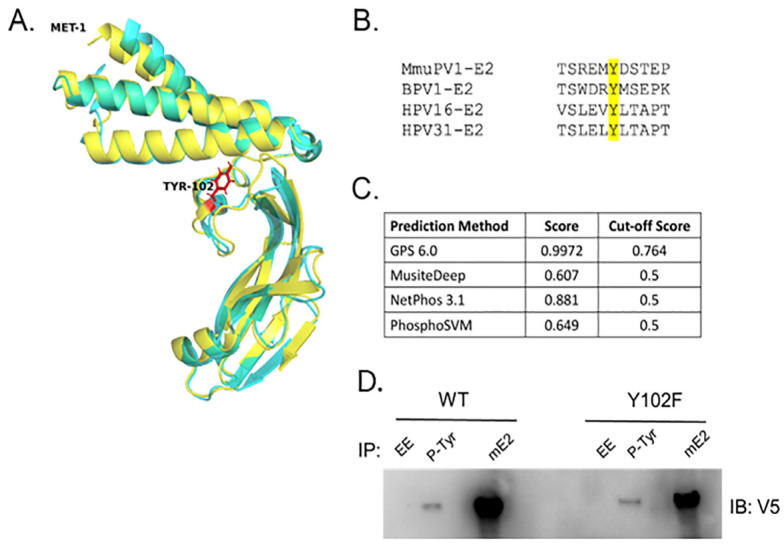
(**A**) Structure alignment between the predicted MmuPV1 E2 TAD (yellow) and the BPV-1 E2 TAD (cyan, PDB: 2JEU). Y102 is identified in red. Structure prediction was performed with I-TASSER-MTD, and alignment was performed in PyMOL. (**B**) Multiple sequence alignment of the TAD sequences from MmuPV1, BPV-1, HPV-16, and HPV-31. Y102 is highlighted in yellow. (**C**) Multiple prediction algorithms indicate that Y102 is likely to be phosphorylated in MmuPV1 E2. (**D**) HEK293TT cells were transfected with V5-E2 WT and Y102F constructs. Forty-eight hours later, lysate was subjected to either EE (non-specific monoclonal control), phospho-tyrosine antibodies (pTyr-1000, Cell Signaling), or MmuPV1 E2 antibodies. Immunocomplexes were blotted with V5 antibodies.

Tyrosine 102 (Y102) is positioned within the fulcrum of the E2 transactivation domain (TAD) of E2. The MmuPV1 E2 structure was predicted using I-TASSER-MTD [16] and aligned with the solved structure of the BPV-1 E2 TAD (PDB: 2JEU, [17]) in PyMOL to demonstrate that the position of this residue is maintained (Figure 1A). This residue is highly conserved across PV types, including several high-risk HPVs (Figure 1B). Our lab has previously identified phosphorylation on Y102 through mass spectrometry analysis of the E2 proteins from BPV-1 [18] and HPV-31 [19]. The PTM prediction tools GPS 6.0 [20], MusiteDeep [21,22,23], NetPhos 3.1 [24], and PhosphoSVM [25] suggested that MmuPV1 E2 Y102 is a potential site of phosphorylation (Figure 1C). To detect tyrosine phosphorylation on the MmuPV1 E2 protein, HEK293TT cells were transfected with V5-tagged WT or Y102F E2 constructs, and the E2 proteins were immunoprecipitated with either antibodies that recognized tyrosine phosphorylation (pTyr) or a mouse monoclonal antibody to MmuPV1 E2 that we generated (Figure 1D). Both the WT and Y102F E2 proteins were detected with the pTyr antibody, indicating that E2 is phosphorylated on tyrosine residues other than Y102. This was not surprising, as we previously identified multiple phosphorylated tyrosine residues on HPV E2 proteins, including at Y131 and Y138.

### 3.2. Y102E Is Transcription and Replication Defective

**Figure 2:**
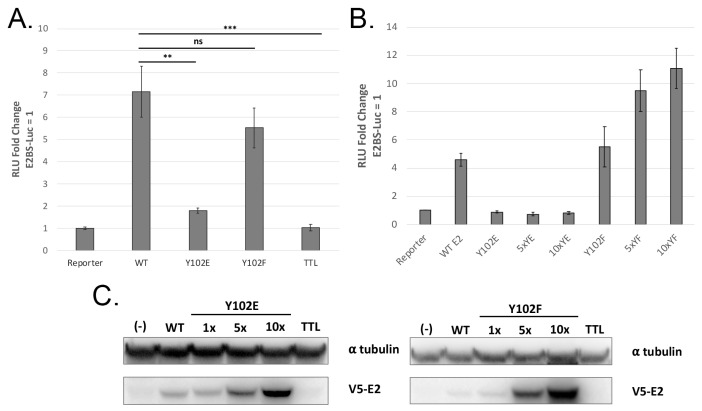
Y102E is incapable of activating transcription. (**A**) Transcriptional activity of Y102 E2 mutants was assessed by luciferase assay. C33-A cells were transfected with an E2-responsive reporter, pGL2-E2BS-Luc, and either WT or mutant E2 expression vectors. Luciferase activity was measured 48h post-transfection. Y102E is defective for transcription activation; Y102F transcription activation is not significantly different from WT E2. (**B**) The transcription assay was repeated with increasing amounts of each Y102 mutant expression vector. Y102F demonstrates dose-dependent transcriptional activation. (**C**) Whole-cell lysates from the overexpression assay in panel B were separated by SDS-PAGE followed by immunoblotting. E2 was blotted with V5, and tubulin was blotted as a loading control. Both Y102 mutants are expressed in a dose-dependent manner. N = 3, * *p* < 0.05, ** *p* < 0.01, *** *p* < 0.001 ns is not significant, paired *t*-test.

To investigate the activities of Y102 in MmuPV1 E2, site-directed mutagenesis was performed using primers to substitute Y102 with glutamate (E), a possible mimic for phosphotyrosine, or phenylalanine (F), a substitution that is not phosphorylated [26]. These Y102 mutants were assessed for their ability to activate transcription from the E2-responsive firefly luciferase reporter pGL2-E2BS-Luc [27]. While Y102F increased transcription comparably to WT E2, Y102E displayed a severe transcriptional defect (Figure 2A). Overexpression of Y102F showed a dose-dependent increase in expression of the reporter, while Y102E protein activity was not rescued (Figure 2B). To ensure that the transcriptional defect of Y102E was not due to poor expression, whole-cell lysates were separated by SDS-PAGE and immunoblotted for V5-E2. Both mutants displayed dose-dependent expression comparable to WT (Figure 2C). We concluded that the transcriptional defect displayed by Y102E was not due to insufficient expression of the mutant protein.

**Figure 3:**
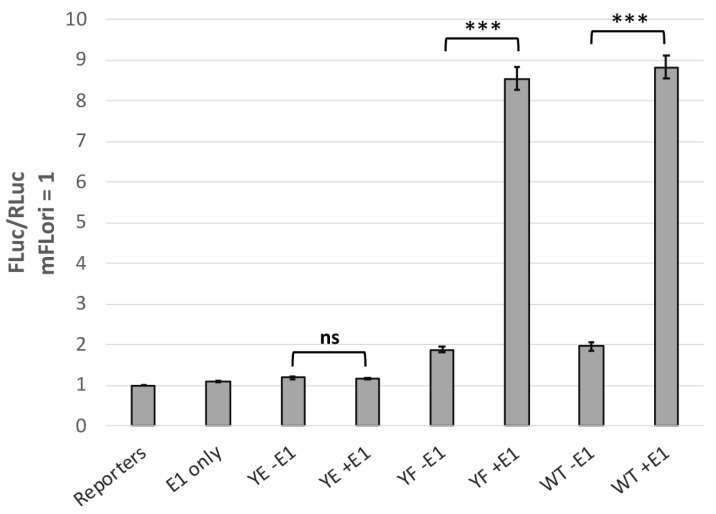
Y102E fails to support transient replication. C33-A cells in a 96-well plate were transfected with 5.6ng mFLori, 1.4ng pRLuc, 28ng pCI-myc-E1 (WT or TTL), and 14ng pCI-V5-E2 (WT or mutant) per well. Firefly luminescence was normalized to renilla luminescence; FLuc/RLuc ratios were then normalized relative to samples without E1 or E2 expression. Replication fold change is presented as the average across three independent biological replicates, each consisting of eight technical replicates. N = 3, * *p* < 0.05, ** *p* < 0.01, *** *p* < 0.001, ns is not significant, paired *t*-test.

In cooperation with E1, E2 is required for viral DNA replication [28,29]. To investigate how phosphorylation at Y102 may affect E1-mediated replication, we utilized the luciferase-based transient replication assay developed by the Archambault lab [11]. C33-A cells were transfected with WT or mutant pCI-V5-E2, pCI-myc-E1, pRLuc, and mFLori, a reporter containing the MmuPV1 LCR downstream of the firefly luciferase gene. Early termination mutants E1 TTL and E2 TTL were used as controls to measure luminescence when one or both replication proteins are absent. When WT and Y102F E2 were expressed in conjunction with E1, both displayed robust transient replication with an FLuc/RLuc signal approximately 8-fold increased over baseline (Figure 3). In contrast, Y102E was incapable of supporting transient replication to any significant degree.

**Figure 4:**
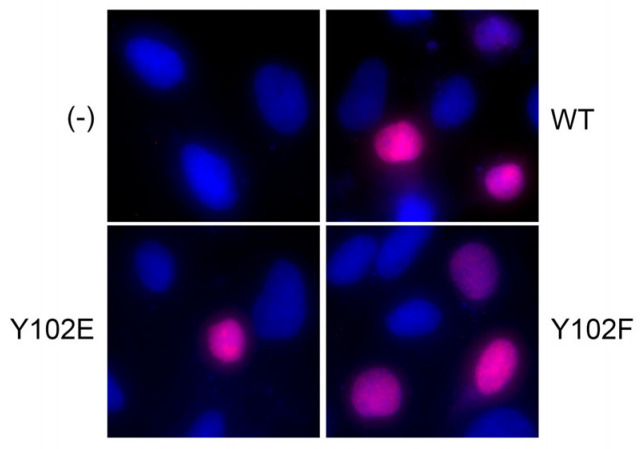
Mutation of Y102 does not affect nuclear localization. CV-1 cells were transfected with 3 μg of WT or Y102 mutant pCI-V5-E2. Cells were fixed and stained 48 h post-transfection. Both Y102E and Y102F display nuclear localization similar to WT E2. V5-E2 (red) DAPI (blue).

E2 proteins localize within the host cell nucleus. One of the putative nuclear localization signals of E2 is located within the TAD near Y102 [30]. Because Y102E was functionally inert, we hypothesized that Y102E may mislocalize to the cytoplasm. Immunofluorescence was performed on CV-1 cells transfected with expression vectors for the V5-tagged Y102 mutants. Untransfected cells were used as the negative control, and cells transfected with the WT V5-E2 vector were used as the positive control. Neither mutant displayed notable cytoplasmic localization (Figure 4), so the defects displayed by Y102E could not be attributed to failure to access the nucleus.

### 3.3. Y102 Affects E2 Protein–Protein Interactions

**Figure 5:**
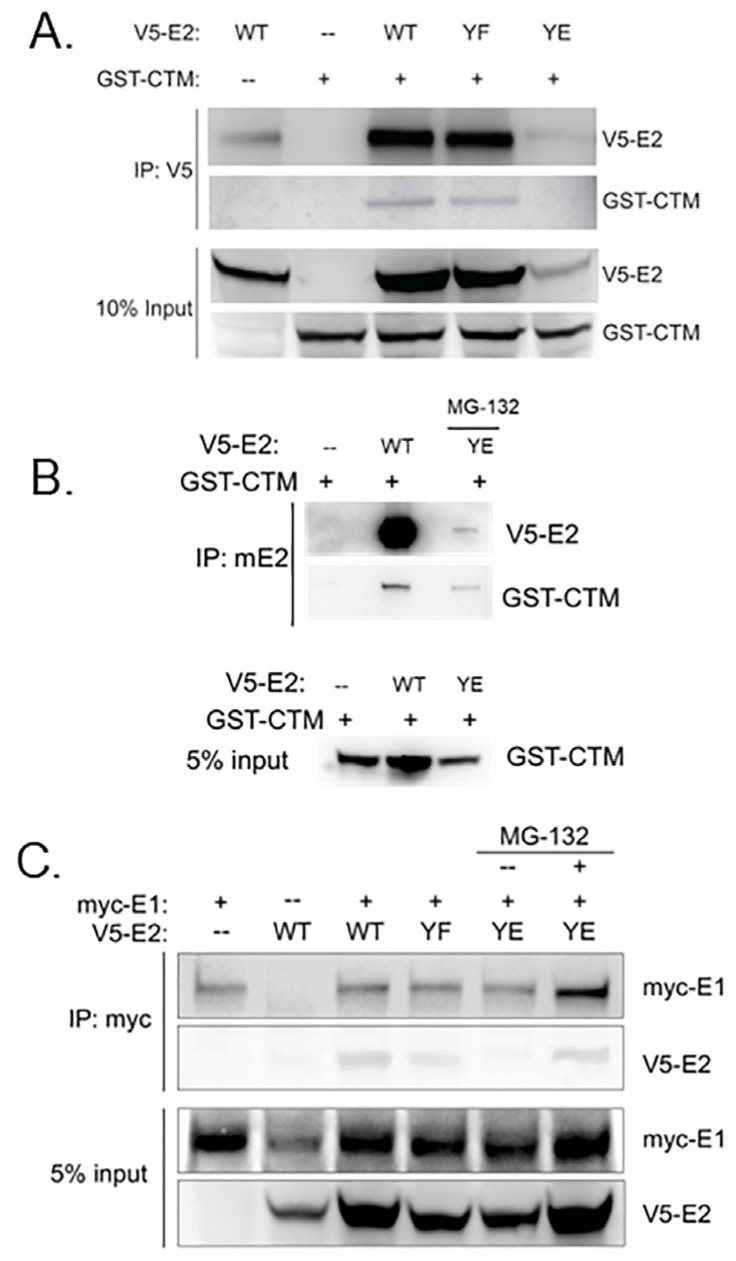
Y102 regulates E2 protein interactions. (**A**) Co-immunoprecipitation of WT and Y102 mutant V5-E2 with GST-tagged Brd4 CTM. The CTM co-IPs with WT and Y102F E2 but not Y102E. Y102E (lane 5) demonstrates decreased protein stability. (**B**) Co-immunoprecipitation of WT and Y102E V5-E2 with GST-tagged Brd4 CTM with MmuPV1 E2 antibodies. The Y012E group was treated with MG-132. (**C**) Co-IP of WT and Y102 mutant V5-E2 with myc-E1. WT and Y102F (lanes 3 and 4) co-IP with E1. Y102E is defective for E1 binding but this interaction is restored in the presence of MG-132 (compare lanes 5 and 6).

E2 proteins interact with multiple viral and host proteins during the papillomavirus life cycle, including bromodomain-containing protein 4 (Brd4), although affinities among HPV types are variable [31]. Work from our lab has previously shown that the phospho-mimetic mutant Y102E in HPV-31 [19] and BPV-1 [18] did not bind the C-terminal motif (CTM) of Brd4. Therefore, we next sought to determine whether mutation at Y102 altered the ability of MmuPV1 E2 to bind the Brd4 CTM. Co-immunoprecipitation experiments were performed with cell lysates expressing a GST-tagged Brd4 CTM fragment and WT or mutant pCI-V5-E2. While both WT and Y102F pulled down the Brd4 CTM, Y102E did not (Figure 5A). Additionally, Y102E displayed decreased protein stability, as demonstrated by relatively lower E2 protein visible in the IP and the input. We treated cells expressing Y102E with 50 µM MG-132 for 5 h prior to lysis and immunoprecipitation and observed that, under these conditions, Y102E interacted with the Brd4 CTM (Figure 5B); however, Y102E protein levels remained lower than WT and Y102F, indicating reduced stability despite complex formation with the CTM.

We next investigated whether the Y102E protein complexes with MmuPV1 E1 [28,29,32,33]. Since Y102E did not induce transient replication, we predicted that this mutant might be unable to bind E1. Whole-cell lysate from HEK293TT cells transfected with myc-tagged E1 and V5-tagged-E2 expression vectors was immunoprecipitated with myc-conjugated magnetic beads to isolate protein in complex with E1. To compensate for the decreased stability of Y102E demonstrated in Figure 5A,B, samples containing Y102E were transfected with 1.5x the amount of WT or Y102F expression vectors. MmuPV1 E2 Y102E retained E1 binding, although their association was observed only when Y102E was overexpressed and with the inclusion of the proteasome inhibitor MG-132 (Figure 5C, compare lanes 5 and 6). Y102F was able to bind E1, which was expected based on previous studies and the results of the transient DNA replication assay (Figure 3).

### 3.4. The E2 Y102F Mutant Genome Is Competent for Establishment and Production of Infectious Virus In Vivo

The in vitro replication data suggested that mutation of Y102 may affect the development of cutaneous disease in vivo. To test this hypothesis, genomes harboring the Y102E or Y102F E2 mutations were prepared as described previously [15]. Athymic hairless mice were anesthetized prior to tail injection with approximately 18 μg of either mutant DNA genome. We have previously confirmed using both WT and mutant genomes can induce cutaneous lesions by 6 months [34,35].

**Figure 6:**
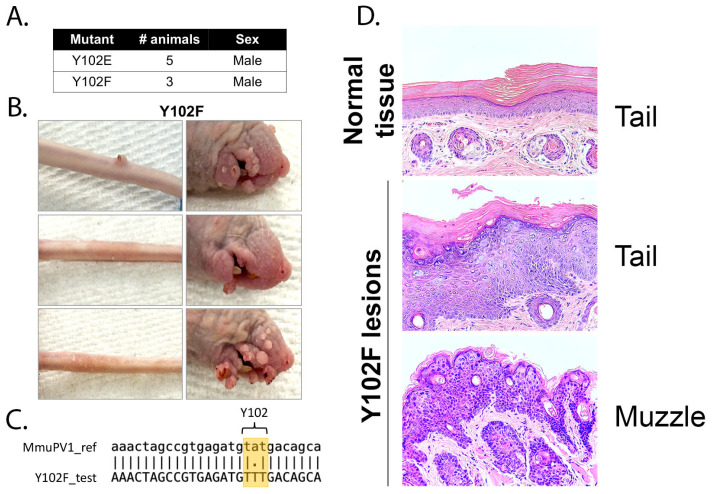
Y102F causes cutaneous disease in mice. (**A**) Table summary of animals used for in vivo experiments. (**B**) Representative images of athymic hairless mice injected with Y102F genomes at the time of sacrifice, approximately 5.5 months post-injection. (**C**) Sequence alignment of the MmuPV1 reference genome and DNA isolated from a Y102F lesion. (**D**) Hematoxylin and eosin (H&E) stained tissue sections from mice injected with Y102F genomes. The top panel is from a section of tail without visible cutaneous lesions. The middle panel is taken from the same animal from a diseased section of tail. The bottom panel is a lesion collected from the muzzle of the same animal. The middle and bottom panels display hyperproliferation characteristic of papillomatous growths.

Mice challenged with Y102F genomes began to develop visible lesions at the site of injection on the tail approximately 3.5 months post-injection. By 5 months post-injection, all Y102F-exposed mice had developed lesions on the tail, and the infection had spread to the muzzle. No mice in the Y102E group developed visible lesions. A summary of these experimental findings is shown in Figure 6A.

At approximately 5.5 months post-injection, all mice were sacrificed, representative images were taken, and tissue was collected for analysis. Mice injected with Y102F displayed florid lesion growth on the muzzle, indicative of secondary infection, while tail lesions had begun to regress (Figure 6B). DNA was isolated from one lesion collected from the Y102F group and subsequently used in PCR reactions to amplify the E1 and E2 open reading frames. Sequencing confirmed that the lesions encoded the Y102F mutation within the E2 ORF (Figure 6C).

Tail and muzzle tissue sections from a Y102F-injected mouse were fixed and embedded in paraffin for histological analysis. Hematoxylin and eosin staining was performed, and representative images were taken (Figure 6D). The top panel was collected from a section of tail lacking any visible lesions and displays normal histologic features. The middle panel is from a section of the same tail with a visible cutaneous lesion proximal to the normal tissue sample. The bottom panel is a lesion isolated from the muzzle of the same mouse. In the middle and bottom panels, hyperkeratosis and papillomatosis can be appreciated. Koilocytes are readily observed in the middle panel.

To further confirm that the E2 Y102F mutation did not interfere with the ability to produce infectious progeny virus, the Y102F mice were removed, and a new batch of naïve mice were placed into this cage. About 2 months post-exposure, the naïve mice began to develop lesions on the muzzle. By 3 months post-exposure, all mice displayed florid papillomatous growths on the muzzle. We conclude that E2 Y102F can replicate in vivo and produce infectious virus.

**Figure 7:**
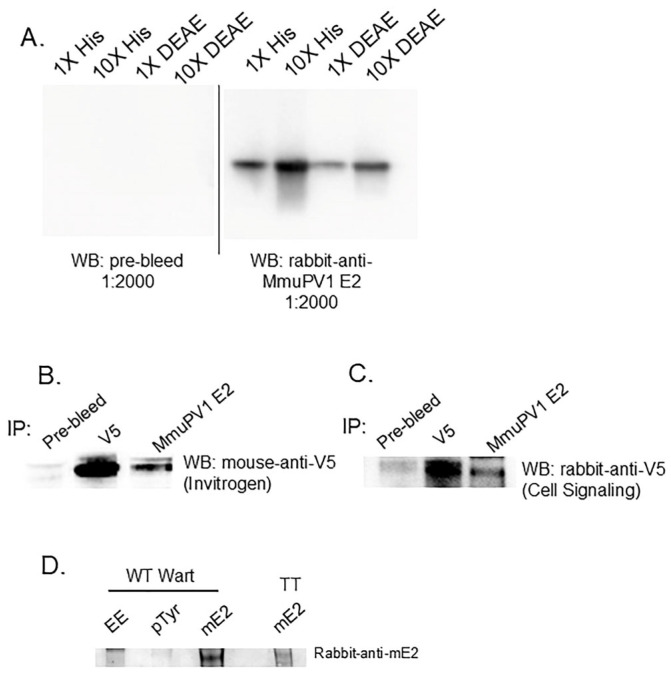
Detection of MmuPV1 E2 protein. (**A**) Recombinant His-MmuPV1 E2 aa 1-221 protein was purified using Ni-beads (His) and/or ion-exchange chromatography (DEAE). Purified His-MmuPV1 E2 aa 1-221 was Western blotted with either pre-bleed or rabbit-anti-MmuPV1 E2 antibodies. HEK293TT cells were transfected with V5-tagged MmuPV1 E2 plasmid. Forty-eight hours later, cells were lysed as whole cell extracts (**B**) or nuclear extracts (**C**), and the MmuPV1 E2 protein was immunoprecipitated with commercial V5 antibodies (Invitrogen) or the rabbit anti-MmuPV1 E2 antibodies. Rabbit pre-bleed was used as a negative control. The presence of MmuPV1 E2 protein was probed using V5 antibodies. (**D**) WT warts were subjected to immunoprecipitation with EE (non-specific IgG control), pTyr, and mouse anti-mE2 antibodies. MmuPV1 E2 expressed in HEK293TT cells was used as a positive control. Immunoblots were blotted with rabbit anti-MmuPV1 E2 antibodies.

To attempt detection of the MmuPV1 E2 protein in the murine warts by Western blot, we used a rabbit antiserum generated to the MmuPV1 E2 transactivation domain (Figure 7A). The antiserum reactivity was confirmed by IP of V5-tagged MmuPV1 E2 protein expressed (Figure 7B,C). WT wart tissue was extracted and immunoprecipitated using EE (non-specific IgG control), pTyr, or the monoclonal MmuPV1 E2 antibody. These blots detected wild-type but not Y102F MmuPV1 E2 protein (Figure 7D). Nonetheless, Y102F expression is indicated by its ability to infect mice and produce infectious virus. We were unable to perform this experiment to detect the Y102E protein because this mutant genome did not induce wart formation in mice.

## 4. Discussion

The papillomavirus (PV) replicative program is tightly regulated. Although the E1 and E2 proteins initiate and coordinate viral genome replication, their activity alone does not lead to lytic infection, suggesting that a mechanism exists to limit genome amplification. This regulation is also dependent on the differentiation state of the host cell [1]. Temporal control of the viral life cycle has primarily been studied using transfected keratinocytes or, more recently, infectious quasiviruses in epithelial cell models. However, the mechanism by which PV switches from initial genome replication to differentiation-dependent amplification and capsid protein expression—observed both in vitro and in vivo—remains unclear.

The E2 protein serves as the central coordinator of the PV life cycle. It binds the viral E1 helicase and interacts with several host cellular factors, including TFIIB, TFIID, AMF-1/GPS2, Brd4, Brm, and TopBP1, among others [27,31,36,37,38,39,40,41]. These interactions are regulated by post-translational modifications (PTMs) of E2, which influence its roles in transcription, replication, and genome partitioning.

Our lab identified several phosphorylated tyrosine residues in E2 proteins across HPV and BPV types. A conserved tyrosine at position 102 is present in many E2 proteins and is a candidate for phosphorylation based on bioinformatic prediction. To examine its role, we generated two E2 mutants: Y102E (a phosphomimetic) and Y102F (a non-phosphorylatable analog). Functional assays showed that the Y102E mutant failed to activate transcription in vitro, whereas Y102F retained full transcriptional activity. These results are consistent with previous findings in BPV-1 E2.

Viral replication requires both E1 and E2 [28,42,43]. E2 recruits E1 to the origin of replication, and mutations that disrupt this interaction impair replication [29,32,33,42,44,45]. In our study, MmuPV1 E2 Y102F bound E1 and supported E1-dependent transient replication. In contrast, the Y102E mutant, despite binding E1 in co-immunoprecipitation assays, was unable to stimulate transient DNA replication. This differs from BPV-1, where Y102E fails to bind E1, but resembles HPV-31, where Y102E binds E1 but shows moderate replication defects. Overall, the MmuPV1 Y102E mutant more closely parallels the behavior of BPV-1 E2 mutants than HPV-31 mutants.

The replication defect associated with Y102E appears to be multifaceted. Brd4, a bromodomain-containing transcriptional regulator [46], interacts with E2 through its transactivation domain (TAD) and modulates both transcription and replication [47,48,49]. Binding to the C-terminal motif (CTM) of Brd4 stabilizes E2 by preventing proteasomal degradation [48,50,51]. We confirmed that both WT and Y102F E2 interact with Brd4-CTM, as demonstrated by GST pulldown assays. For the Y102E mutant, Brd4 interaction was only detectable following proteasome inhibition with MG-132, indicating that Y102E is rapidly degraded. This suggests that Brd4 binding may be reduced but not entirely lost; however, this low level of Brd4 binding is not sufficient for Y102E stabilization—structural analysis supports this conclusion.

Although Y102 lies in the fulcrum of the E2 TAD and not on the same helices as the Brd4 interaction residues R37 and I73, the CTM crystal structure used previously did not cover this region [49]. AlphaFold [52] modeling revealed that replacing tyrosine with glutamic acid at position 102 does not grossly alter E2 structure. This supports our biochemical data indicating that global misfolding or complete disruption of Brd4 binding is unlikely to be the sole cause of Y102E instability. The precise mechanism underlying the instability of Y102E in cell culture remains unknown.

While monolayer cell culture is the standard method for studying PV replication, it does not fully recapitulate the in vivo viral life cycle. To address this, we evaluated the Y102 mutants using the MmuPV1 mouse model. Only a few prior studies have investigated early gene mutations in this system [35,53,54]. Our data show that Y102 is not essential for infectivity, as the Y102F mutant successfully induced papillomas and produced infectious virus in mice. This indicates that phosphorylation at Y102 is not required for viral infectivity, pathogenesis, and virion assembly.

We previously hypothesized that substitution of Y102 with a negatively charged residue might mimic a regulatory state that suppresses transcription and replication early in infection, while the non-phosphorylatable Y102F mutant might drive unchecked replication and a lytic phenotype [18]. However, this was not observed in vivo using MmuPV1. The Y102F viral genome behaved similarly to wild-type, producing typical warts and infectious virions. In contrast, the Y102E mutant genome failed to replicate efficiently in vitro and was not maintained in vivo. These results lead us to conjecture that Y102 phosphorylation interferes with the interaction of E2 with a cellular factor necessary for E2 protein stabilization and function. This suggests that post-translational modification of Y102 may modulate E2 stability or a key host–virus interaction required for establishing a productive infection.

## Data Availability

The data presented in this study are available upon request from the corresponding author.
